# PRF Lysates Modulate Chemokine Expression in Oral Squamous Carcinoma and Healthy Epithelial Cells

**DOI:** 10.3390/bioengineering11080746

**Published:** 2024-07-24

**Authors:** Zohreh Afradi, Layla Panahipour, Salman Abbas Zadeh, Reinhard Gruber

**Affiliations:** 1 Department of Oral Biology, University Clinic of Dentistry, Medical University of Vienna, 1090 Vienna, Austria; zohreh_afradi@yahoo.com (Z.A.); layla.panahipour@meduniwien.ac.at (L.P.); 2 Epitome GmbH, 1100 Vienna, Austria; salman.abbaszadeh@gmx.at; 3 Department of Periodontology, School of Dental Medicine, University of Bern, 3010 Bern, Switzerland; 4 Austrian Cluster for Tissue Regeneration, 1200 Vienna, Austria

**Keywords:** platelet-rich fibrin, PRF, oral squamous carcinoma cells, HSC2, TR146, oral epithelial cells, chemokines, inflammation

## Abstract

Platelet-rich fibrin (PRF), originally used to support soft tissue healing, is also considered a therapeutic option for treating oral lichen planus and leukoplakia. The progression from the two premalignant lesions to the aggressive malignant oral squamous cell carcinoma involves an inflammatory process linked to chemokine expression. Thus, there is a rationale for studying how PRF modulates the expression of chemokines in oral squamous carcinoma cells. To this aim, we expose the oral squamous carcinoma cell line HSC2 to IL1β and TNFα either alone or in the presence of lysates obtained from solid PRF membranes. We report here that in HSC2 cells, PRF lysates significantly reduce the forced transcription of chemokines, e.g., CXCL1, CXCL2, CXCL8, CXCL10, and CCL5. Moreover, PRF lysates attenuate the nuclear translocation of p65 in HSC2 oral epithelial cells when exposed to IL1β and TNFα. PRF lysates further reduce chemokine expression provoked by poly:IC HMW. Even though less pronounced, PRF lysates reduce IL1β- and TNFα-induced chemokine expression in TR146 cells. In primary oral epithelial cells, however, PRF lysates increase the basal expression of CXCL1, CXCL2 and CXCL8. Thus, PRF can exert a biphasic effect on chemokine expression in oral squamous cell carcinoma cell lines and primary oral epithelial cells. These findings suggest that PRF may reduce inflammation in a malignant environment while provoking an immunological response in healthy oral epithelium.

## 1. Introduction

Oral mucosa plays a role as a barrier in the oral cavity protecting the underlying tissues from mechanical damage and noxious agents of hazardous and damaging influence [1]. The oral mucosa encompasses a stratified squamous epithelium, the oral epithelium, and the underlying connective tissue of the lamina propria [1]. The primary cell compartments in oral mucosa comprise epithelial, endothelial, fibroblast, and immune cells [2]. The integrity of the oral epithelium barrier is critical for maintaining oral health and includes the nonkeratinized and keratinized epithelium of the lining and masticatory mucosa, respectively. Epithelial cells also define the sulcular and junctional epithelium defending and sealing the tooth towards the oral cavity, steadily renewed by stem cells [3]. Thus, oral epithelial cells fulfill the difficult task of maintaining oral health but cannot eternally preclude the development of inflammatory lesions. For instance, chronic oral mucositis increases the risk for premalignant lesions such as oral leukoplakia [4] and oral lichen planus [5], which may even undergo malignant transformation into aggressive oral squamous cell carcinoma [6]. There is thus a demand to decrease local inflammation.

Oral mucositis in its severe form is a common devastating obstacle of cancer treatment, particularly chemotherapy and radiation [7]. Impaired epithelial integrity causes pain, inability to eat, and increased risk of infection-producing inflammation [7]. While gingivitis and peri-implant mucositis can be prevented and treated by good oral hygiene and by professional cleaning, in periodontitis and peri-implantitis, the junctional epithelium and the soft tissue sealing, respectively, are severely deteriorated [8,9]. Defects are homing the microbial biofilms, releasing virulence factors, further triggering the inflammatory response of the innate immune system [10,11]. The vicious cycle of a destructive inflammatory process affecting the soft and hard tissues surrounding teeth and dental implants must be interrupted. The inflammatory process is also linked to the progression of oral leukoplakia and oral lichen planus [4,5]. There is consequently a demand for anti-inflammatory treatments to ease oral mucositis symptoms and recover from oral leukoplakia and oral lichen planus—potentially avoiding its transformation into oral squamous cell carcinoma.

Apart from the classical inflammatory macrophages and lymphocytes, oral epithelial cells contribute to the vicious cycle of inflammation by releasing cytokines and chemokines [2]. Oral epithelial cells in periodontitis are a moderate source of chemokines, including CXCL1, CXCL3, CXCL5, CXCL16, CCL28, and CCL20, together signaling molecules attracting neutrophils and other immune cells towards the site of inflammation [2]. Moreover, nuclear staining of p65 suggesting inflammatory NFkB signaling was observed in the pocket epithelium of periodontitis tissue [12]. Apart from periodontitis, it is particular chemokines that are important for promoting tumor progression in oral squamous cell carcinoma. For instance, CXCL1 is an inflammatory mediator associated with metastasis of oral squamous cell carcinoma [13]. Porphyromonas gingivalis induces CXCL2, promoting inflammation and oral squamous cell carcinoma progression [14]. Salivary and tissue CXCL8 is relevant in early diagnosis and prognosis of oral squamous cell carcinoma [15,16,17]. CCL5 antagonists reduce neck metastasis [18]. Moreover, oral squamous cell carcinoma cell lines, including HSC2, respond to inflammatory cytokines IL1β and TNFα with an increased expression of chemokines such as CXCL1, CXCL2, CXCL8, and CCL5 [19]. There is thus solid evidence that oral squamous cell carcinoma cells are a source of chemokines driving inflammation and, indirectly, their malignancy. For instance, single cell RNAseq revealed chemokine signaling pathways to be active in epithelial cells of oral squamous cell carcinoma [20]. Strategies to decrease or at least not to increase the expression of chemokines are thus of potential clinical relevance. To this aim, we implemented HSC2 and TR146 oral squamous cell carcinoma cell lines serving as potential bioassays to evaluate chemokine expression.

Platelet-rich fibrin (PRF) is a plasma-derived autologous preparation of centrifuged venous blood [21]. Depending on the tubes used for blood collection, the coagulation is initiated by a rough glass surface or reduced by a hydrophobic plastic surface, cumulating in solid and liquid PRF, respectively [22,23]. Depending on the gravity force and time, as well as the selection of fixed angle or swing-out centrifuges, PRF can be modified—but overall, the concept is to generate a coagulated fibrin-rich matrix where platelets and leucocytes accumulate [24]. Regardless of whether it is solid or liquid PRF, the respective lysates have an intense anti-inflammatory activity in murine bioassays; for instance, solid PRF and liquid PRF, as well as lysates from coagulated blood, reduce the in vitro inflammatory response of macrophages [25,26,27,28] and mesenchymal cells [29]. Moreover, there was a trend that solid PRF lysates reduced the CXCL8 expression in HSC2 oral epithelial cells [29], and other in vitro studies support the use of oral epithelial cells as a target for PRF [30]. Recent data, however, showed that in primary gingival fibroblasts, PRF lysates increase CXCL8 expression [31]. We now refine this research line towards extending the spectrum of chemokines and implementing HSC2 and TR146 oral squamous cell carcinoma and primary human oral epithelial cells into the present study design.

## 2. Materials and Methods

### 2.1. Cell Culture

The oral squamous cell carcinoma cell lines HSC2 and TR146 were obtained from the Health Science Research Resources Bank (Sennan, Japan) and the European Collection of Authenticated Cell Cultures (ECACC; Salisbury, UK), respectively. The cells were expanded in growth Dulbecco’s modified Eagle’s medium (DMEM, Sigma-Aldrich, St. Louis, MO, USA), 10% fetal calf serum (Bio&Sell GmbH, Nuremberg, Germany) and 1% antibiotic–antimycotic solution (Sigma Aldrich, St. Louis, MO, USA). Cells were seeded at 2.5 × 10^5^ cells/cm^2^ into 24-well plates the day before being exposed to respective treatments. Primary oral epithelial cells were isolated from human gingiva obtained at the occasion of wisdom tooth extraction after patients provided their informed and written consent (#631/2007) as described recently [32]. In brief, epithelial sheets were separated from connective tissue after overnight dispase II (2.4 U/mL; Roche, Mannheim, Germany) treatment. Trypsin digestion was used to prepare a single-cell suspension (Lonza, Walkersville, MD, USA) that was expanded in a keratinocyte growth medium (PromoCell, Heidelberg, Germany). Cells of the first passage were used for the experiments.

### 2.2. Preparation of Solid PRF Lysates

After approval of the Ethics Committee of the Medical University of Vienna (#1644/2018) and signing the informed consent form, we collected the venous blood from four healthy female volunteers, 26–38 years of age, using plain glass tubes (Bio-PRF, Venice, FL, USA). These blood coagulation tubes were centrifuged at 700× *g* for 8 min with universal swing-out rotor (Z 306, Hermle, Universal Centrifuge, Wehingen, Germany). The solid PRF was separated from the remaining red clot and compressed between two layers of dry gauze. The resulting PRF membranes were transferred to a serum-free medium (1 cm PRF membrane/mL serum free medium) and exposed to two freeze–thawing cycles and sonication (Sonopuls 2000.2, Bandelin Electronic, Berlin, Germany). To prepare liquid PRF, venous blood was collected in plastic tubes (“No Additive“, Greiner Bio-One GmbH, Kremsmünster, Austria) and centrifuged at 700× *g* for 8 min to separate the platelet-poor plasma (PPP) from the buffy coat fraction (BC). Both fractions of liquid PRF underwent two freeze–thawing cycles and sonication. All lysates were centrifuged at 15,000× *g* for 10 min, and the supernatant was filtered sterilized and stored at −80 °C before cell exposure.

### 2.3. Reverse Transcription Quantitative Real-Time PCR (RT-qPCR) and Immunoassay

After overnight stimulation of the cells with 30% PRF lysate, 10% PPP and 10% BC, total RNA was isolated with the ExtractMe total RNA kit (BlirtS.A., Gda’nsk, Poland) followed by reverse transcription (LabQ, Labconsulting, Vienna, Austria) and polymerase chain reaction (LabQ, Labconsulting, Vienna, Austria) on a CFX Connect™ Real-Time PCR Detection System (Bio–Rad Laboratories, Hercules, CA, USA). The primer sequences for CXCL8, CXCL1, and CXCL2 [19] and CXCL10, CCL5 and GAPDH [33] are published. The mRNA expression was calculated by normalizing target gene expression to the housekeeping gene GAPDH using the ΔΔCt method. According to the manufacturer’s instructions, lysates of HSC2 cells and supernatants of primary oral epithelial cells were analyzed for CXCL8 by immunoassays (R&D Systems, Minneapolis, MN, USA).

### 2.4. Immunofluorescence Analysis

Immunofluorescence analysis of p65 nuclear translocation was performed in HSC2 cells. The cells were plated on Millicell EZ slides (Merck KGaA, Darmstadt, Germany) at a 15,000 cell/cm^2^ density and subjected to serum starvation overnight with 30% PRF lysates. To induce inflammation, the cells were exposed to IL1β and TNFα for 40 min. The cells were fixed with 4% paraformaldehyde, blocked with 1% bovine serum albumin (Sigma-Aldrich, St. Louis, MO, USA), and permeabilized with 0.3% Triton X-100 (Sigma-Aldrich, St. Louis, MO, USA). We used anti-p65 antibodies (IgG, 1:400, Cell Signaling Technology, #8242, Danvers, MA, USA) at 4 °C overnight. Detection was performed with a goat anti-rabbit Alexa 488 secondary antibody (CS-4412, 1:800, Cell Signaling Technology). Images were captured on a fluorescence microscope with the DAPI-FITC dual excitation filter block (Echo Revolve fluorescence microscope, San Diego, CA, USA).

### 2.5. Statistical Analysis

All experiments were performed independently at least four times. Statistical analyses were performed with ratio paired *t*-tests and Friedmann test for when indicated. Analyses were performed using Prism v.9 (GraphPad Software; San Diego, CA, USA). Significance was set at *p* < 0.05 and *p*-values are reported. Data are presented as scatter blots with median.

## 3. Results

### 3.1. PRF Lysates Suppress IL1β- and TNFα-Induced Chemokine Expression in HSC2 Cells

**Figure 1:**
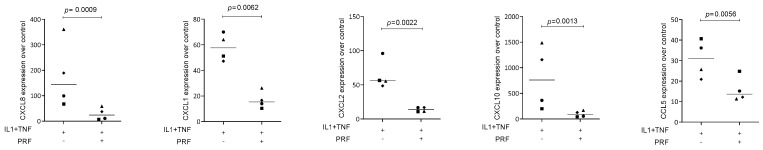
PRF lysates suppress IL1β- and TNFα-induced chemokine expression in HSC2 cells. The oral squamous cell carcinoma cell line HSC2 was exposed to IL1β and TNFα in the presence or absence of PRF lysates. Gene expression analysis was performed the following day. Data points represent four independent experiments. Data were normalized to untreated control cells with x-fold changes compared to the untreated cells. The statistical analysis was based on the ratio paired *t*-test and *p*-values reported.

**Figure 2:**
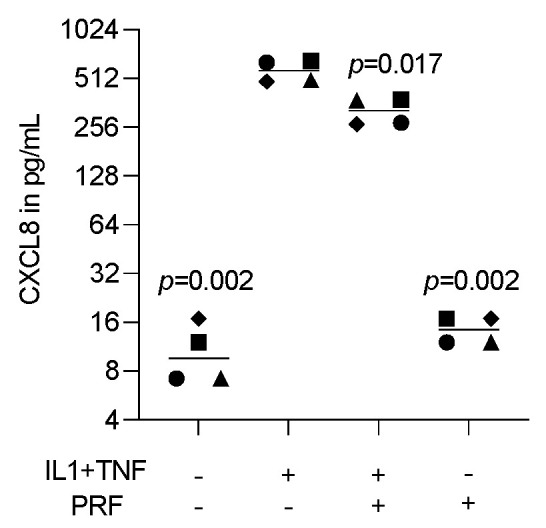
PRF lysates suppress IL1β- and TNFα-induced CXCL8 protein in HSC2 cells. The oral squamous cell carcinoma cell line HSC2 was exposed to IL1β and TNFα in the presence or absence of PRF lysates. A CXCL8 immunoassay from HSC2 cell lysates was performed the following day. Data points represent four independent experiments. Data show CXCL8 in pg/mL of the cell lysates. The statistical analysis was based on the ratio paired *t*-test and *p*-values reported.

To assess the effect of lysates prepared from solid PRF, HSC2 cells were exposed to IL1β and TNFα before chemokine expression was analyzed. We recently identified a chemokine panel consisting of CXCL1, CXCL2, CXCL8, CXCL10, and CCL5 to be highly upregulated by IL1β and TNFα in HSC2 cells [19]. When HSC2 cells were exposed to PRF lysates, the forced expression of all five chemokines was significantly reduced (Figure 1). Support for these findings comes from immunoassay using the lysates of IL1β- and TNFα-treated HSC2 cells exposed to PRF lysates; in this setting, PRF lysates significantly lowered the CXCL8 levels in the cell lysates (Figure 2). Taken together, lysates prepared from solid PRF can reduce IL1β- and TNFα-induced chemokine expression in HSC2 cells.

### 3.2. PRF Lysates Suppress Induced Chemokine Expression in TR146 Cells

**Figure 3:**
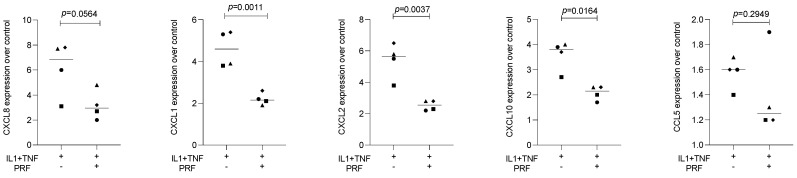
PRF lysates suppress IL1β- and TNFα-induced chemokine expression in TR146 cells. The oral squamous cell carcinoma cell line TR146 was exposed to IL1β and TNFα in the presence or absence of PRF lysates. Gene expression analysis was performed the following day. Data points represent four independent experiments. Data were normalized to untreated control cells with x-fold changes compared to the untreated cells. The statistical analysis was based on the ratio paired *t*-test and *p*-values reported.

To support the observation with HSC2 cells, we implemented TR146, another oral epithelial cell line [34]. When TR146 cells were exposed to IL1β and TNFα, the chemokines CXCL1, CXCL2, CXCL8, and CXCL10 but not CCL5 were significantly increased indicating that TR146 can also serve as a bioassay to test the anti-inflammatory activity of PRF lysates (Figure 3). We avoided immunoassay of the supernatant because TR146 cells spontaneously produced high levels of CXCL8, and the IL1β- and TNFα-induced CXCL8 expression was relatively low [35]. Thus, on the transcriptional level, TR146 cells confirm the impact of PRF lysates to diminish IL1β- and TNFα-induced chemokine expression.

### 3.3. Solid PRF Lysates Reduced the Cytokine-Induced Nuclear Translocation of p65 in HSC2 Cells

**Figure 4:**
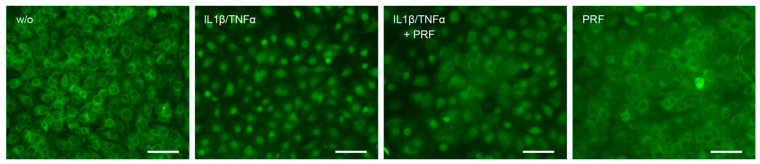
PRF lysates reduced IL1β- and TNFα-induced p65 translocation in HSC2 cells. The oral squamous cell carcinoma cell line HSC2 was preexposed to 30% PRF lysates overnight before IL1β and TNFα were added for 45 min. Immunostaining indicated that PRF lysates reduced IL1β- and TNFα-induced p65 translocation in HSC2 cells. The scale bars represent 100 µm.

To confirm the anti-inflammatory activity of solid PRF lysates, we performed an immunofluorescent analysis of p65 nuclear translocation in HSC2 cells. IL1β and TNFα caused an apparent p65 nuclear staining that was considerably lowered by PRF lysates (Figure 4). Thus, HSC2 cells respond to IL1β and TNFα by p65 nuclear translocation, which is attenuated by solid PRF lysates.

### 3.4. Solid PRF Lysates Suppress Poly(I:C) HMW-Induced Chemokine Expression in HSC2 Cells

**Figure 5:**
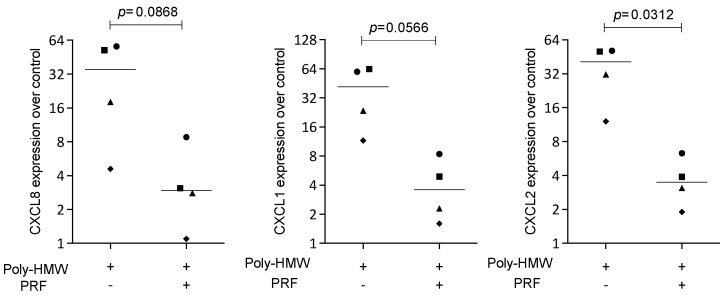
PRF lysates suppress IL1β- and TNFα-induced chemokine expression in HSC2 cells. The oral squamous cell carcinoma cell line HSC2 was exposed to poly(I:C) HMW in the presence or absence of PRF lysates. Gene expression analysis was performed the following day. Data points represent four independent experiments. Data were normalized to untreated control cells with x-fold changes compared to the untreated cells. The statistical analysis was based on the ratio paired *t*-test and *p*-values reported.

To confirm the findings observed for the IL1β- and TNFα-induced inflammation, HSC2 cells were exposed to poly(I:C) HMW, the agonist of TLR3, in the presence or absence of solid PRF lysate. Lysates of PRF reduced the expression of CXCL1, CXCL2, and CXCL8 provoked by poly(I:C) HMW in HSC2 cells (Figure 5). Overall, these findings suggest that the reduction in chemokine expression by PRF lysates can be extended towards TLR-3 agonists in HSC2 oral epithelial cells.

### 3.5. Liquid PRF Failed to Suppress Cytokine-Induced Chemokine Expression in HSC2 Cells

**Figure 6:**
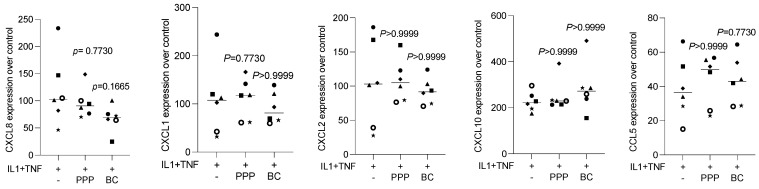
PPP and buffy coat lysates had no impact on IL1β- and TNFα-induced chemokine expression in HSC2 cells. The oral squamous cell carcinoma cell line HSC2 was exposed to IL1β and TNFα in the presence or absence of PPP and buffy coat (BC) lysates. Gene expression analysis was performed the following day. Data points represent independent experiments. Data were normalized to untreated control cells with x-fold changes compared to the untreated cells. The statistical analysis was based on the Friedmann test, and *p*-values were reported.

To understand whether the preparation of liquid PRF exerts anti-inflammatory activity, HSC2 cells were exposed to IL1β and TNFα, with and without lysates of PPP and the buffy coat. We show here that in the presence of lysates of PPP and the buffy coat, the IL1β- and TNFα-induced expression of CXCL1, CXCL2, CXCL8, CXCL10, and CCL5 in HSC2 cells was not considerably changed (Figure 6). Thus, lysates prepared from liquid PPP and the buffy coat are not capable of reducing IL1β- and TNFα-induced chemokine expression in HSC2 cells.

### 3.6. PRF Lysates Increased CXCL8, CXCL1, and CXCL2 Expression in Oral Epithelial Cells

**Figure 7:**
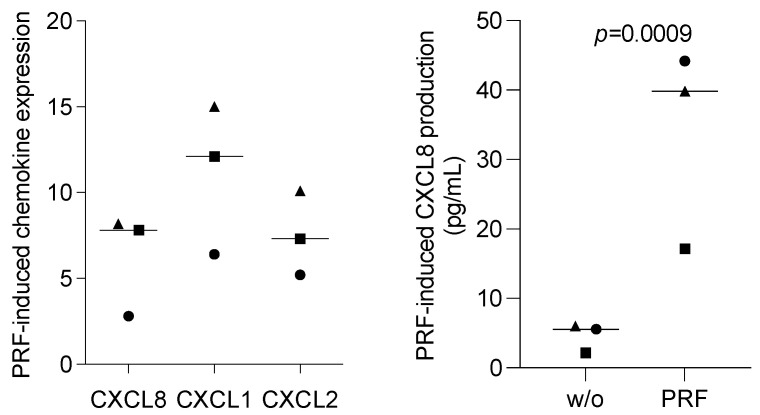
PRF lysates enhanced the CXCL8 production of primary oral epithelial cells. Primary oral epithelial cells were exposed to PRF lysates. Gene expression analysis was performed the following day. Data were normalized to untreated control cells with x-fold changes compared to the untreated cells (left panel). The supernatant was harvested and subjected to immunoassay (right panel). Data points represent three independent experiments. The statistical analysis was based on the ratio paired *t*-test and *p*-values.

To relate the findings observed with the oral squamous carcinoma cell lines to those observed with primary cells, isolated human oral epithelial cells were exposed to PRF lysates. Interestingly, and in contrast to our observations obtained with HSC2 and TR146 cells, PRF lysates enhanced the basal expression of CXCL8, CXCL1, and CXCL2 in primary epithelial cells (Figure 7). Thus, PRF lysates can cause human oral epithelial cells to increasingly express chemokines, which is in contrast to what we observed with the oral squamous carcinoma cell lines.

## 4. Discussion

The present research was driven by the evidence that healthy non-transformed oral epithelial cells contribute to the vicious cycle of periodontal inflammation by their release of cytokines and chemokines [2], for instance, CXCL1, CXCL3, CXCL5, CXCL16, CCL28, and CCL20, together attracting neutrophils and other immune cells towards the site of inflammation [2]. Apart from periodontitis, it is particular chemokines being essential for promoting tumor progression in oral squamous cell carcinoma. For instance, CXCL1 [13], CXCL2 [14], CXCL8 [15,16,17], and CCL5 [18] are all related to the progression and diagnosis of oral squamous cell carcinoma. Moreover, oral squamous cell carcinoma cell lines, including HSC2, respond to inflammatory cytokines IL1β and TNFα with an increased chemokine expression, including CXCL1, CXCL2, CXCL8, and CCL5 [19]. There is, thus, solid evidence that oral squamous cell carcinoma cells are a source of chemokines driving inflammation and perhaps tumor progression. Therapeutic strategies may thus be established to reduce or at least not increase the expression of chemokines. Our first significant finding is that PRF lysates lower the IL1β- and TNFα-induced expression of chemokines by HSC2 and TR146 cells, which is of potential clinical relevance in the context of oncology. This observation obtained with malignant cells becomes even more exciting when considering the second main observation, namely that in healthy oral epithelial cells PRF lysates increased chemokine expression, suggesting PRF to be a driver of healthy oral mucosa innate immunity.

If we relate our findings to those of others, we can acknowledge research showing that oral squamous cell carcinoma cell lines other than HSC2 and TR146 are a source of chemokines, e.g., IL1 enhanced CXCL1 expression in the dysplastic oral keratinocytes and cell lines TW2.6 and OC3 [36]. Moreover, IL1 regulates chemokines, including CXCL1, CXCL8, CCL5, and CXCL10 in normal human oral keratinocytes [37,38]. Also, TNFα induces chemokines, including CXCL8, CXCL10, and CCL5 in human foreskin keratinocytes [39]. The present data further support our previous study showing that HSC2 responds to inflammatory cytokines IL1β and TNFα with an increased chemokine expression which CXCL1, CXCL2, CXCL8, and CCL5 [19]—and in vivo, chemokines including CXCL1 and CXCL8 are linked to oral tongue squamous cell carcinomas [40,41,42,43] and orolaryngeal carcinoma cells [44]. Moreover, a systematic review suggests a role of inflammatory mediators as potential markers, including CXCL1, for the diagnosis and prognosis of oral squamous cell carcinoma metastasis [13]. The present findings support the value of bioassays with HSC2 and TR146 to serve as a mode to study the potential modulation, presumably reduction, of chemokine expression by PRF lysates.

The assumption that PRF lysates will reduce chemokine expression in HSC2 and TR146 is based on previous experiments showing that PRF lysates lower the induced inflammatory response on murine primary macrophages and RAW 264.7 cells [27,28], as well as murine ST2 stromal cells [29]. However, the present research is the first to show such effects in a human epithelial lineage cell model. HSC2 cells serve as a model to show the anti-inflammatory properties of short-chain fatty acids, most of all propionate and butyrate [45] and Z-VAD-FMK (Z-VAD), a pan-caspase inhibitor [33], and the P2 purinergic receptor antagonists [46]. However, so far, the anti-inflammatory activity of PRF lysates has not been shown in cells of human origin. The novelty here is that PRF lysates significantly lower the forced expression of chemokines in human HSC2 and TR146 cells, an activity further supported by the reduced p65 nuclear translocation. Unexpectedly, however, lysates from PPP and buffy coat, with their anti-inflammatory activity in mouse stromal cells [29] and macrophages [27], failed to reduce the chemokine expression of HSC2 cells. These findings support our preliminary findings that solid PRF lysates but not lysates of PPP or the buffy coat reduced IL6 expression of HSC2 in the presence of TNFα and IL1β [29]. It is unclear at the moment why PRF lysates but not the equivalent preparations of PPP or buffy coat are capable of reducing in vitro chemokine expression in tumor cells.

Clinical tumor progression and metastasis are affected by a complex environment that includes an accumulation of inflammatory cytokines such as IL1β and TNFα that, in turn, target the tumor cells. The tumor cells then increase the release of chemokines, attracting tumor-associated neutrophils and other cells that may, in turn, contribute to tumor progression and metastasis [47,48]. Oral neutrophils attracted by the chemokines produced by the malignant cells are considered “underestimated players in oral cancer” [49]. On the other hand, local therapies, including PRF, are widely used to treat oral wound healing and bone regeneration; at least the PRF membranes come in contact with epithelial cells of the oral mucosa—and ideally, PRF does not support the transformation of oral squamous cell carcinomas. However, apart from the therapeutic concept, PRF may represent a pathological environment as, for instance, platelets can increase the invasiveness of cancer cell lines [50,51], and injection of platelets stimulates the formation of metastatic cancer nodules [52]. In general, platelets alter epithelial tumor cell attributes to drive metastasis [53]. Moreover, radiation and chemotherapy are critically linked to mucositis, and PRF might support wound healing under these circumstances [54]. In addition, PRF might target oral epithelial cells in precancerous lesions such as oral leukoplakia [55] and oral lichen planus [56]. Nevertheless, the present research based on HSC2 and TR146 cells [57,58] suggests that PRF may reduce the inflammatory environment of tumor cells and, at least theoretically, reduce the transformation of oral squamous cell carcinomas and the concomitant tumor progression and metastasis. However, these in vitro findings are a primer and should not be interpreted toward the prevention or therapy on oncological situations.

Primary oral epithelial cells, in sharp contrast to the tumor cell lines, show an opposite response. Here, PRF lysates provoked a significant increase in CXCL8, CXCL1, and CXCL2 expression. Thus, the question arises as to why PRF lysates have a biphasic activity, reducing and increasing chemokine expression in HSC2 and primary oral epithelial cells, respectively. This is potentially a clinically relevant observation, and it leads to the speculation that tumor cells and non-transformed primary cells respond differently to PRF. Therefore, we may raise the hypothesis that PRF and presumably also the blood clot itself can drive CXCL8 and other chemokines to be expressed by healthy oral epithelial cells. Moreover, PRF has become a widely accepted clinical option to support the healing of oral soft tissue, including periodontal regeneration, recession coverage [59,60], and socket preservation [61]. Another clinical application is linked to sinus augmentation, where PRF membranes may cover injured sinus mucosa [62] or prepare sticky bone together with liquid PRF [63,64]. Impressive is the success of the application that supports the healing of diabetic ulcers [65] and for dermatological conditions, including androgenetic alopecia, periorbital rejuvenation, and temporary filler material [66]. Thus, from a clinical perspective, epithelial cells come into contact with PRF and may modulate the chemokine expression.

Moreover, surgical interventions initiate the evolutionary sequence of wound healing that starts with a transient inflammation [67], and in pathological situations of chronic inflammation, this scenario is not resolved [68], causing all kinds of katabolic events leading to tissue destruction [69]. Not surprisingly, the inflammation-related expression of chemokines by the epithelial cells is a critical factor in immunity, and single-cell RNAseq of periodontitis gingiva identified epithelial cells to be a source of chemokines, thus capable of attracting neutrophils but also antigen-presenting cells and lymphocytes [2]. This observation is supported by findings from a diabetic mouse model [70]. Considering that oral epithelial cells become a source of chemokine under inflammatory conditions, our in vitro findings that solid PRF is a potent suppression of chemokine expression in oral epithelial cell lines seem important. Our results open the door for future research to refine our understanding of cell specificity in the response of malignant and non-transformed cells to PRF with the overall goal to identify the ideal indication for a local PRF therapy that is based on the understanding of the cell response, with particular emphasis on immunological aspects of healthy and diseased oral epithelium.

These conclusions should be drawn keeping the study limitations in mind. We have to admit that in vitro observation should be interpreted carefully in the clinical context, as we are limited to descriptive information, namely that oral squamous cell carcinoma cell lines TR146 and HSC2 behave differently than primary oral epithelial cells in the contact of how PRF modulates and even initiates chemokine expression. If also in a clinical scenario, PRF distinguishes tumor and healthy epithelial cells on chemokine expression, and the respective potential clinical impact leads to a refined hypothesis. Future research should consider the impact of PRF on epithelial cell expression changes using a single-cell RNAseq approach as reported for periodontitis [2]. Moreover, single-cell RNAseq highlighted chemokine signaling pathways to be functionally enriched in epithelial cells of oral squamous cell carcinoma [20]. Perhaps it will be possible to trace back tissue homeostasis, tissue destruction, and malignant transformation to the chemokine expression by the epithelial cell lineage, with and without the presence of PRF or simply the blood clot.

## Data Availability

All raw data are available upon request.
